# Non-ICANS neurotoxicities CD19-directed CAR T-cell therapy and the emergence of movement and neurocognitive treatment-emergent adverse events: a case report

**DOI:** 10.3389/fimmu.2026.1749587

**Published:** 2026-02-16

**Authors:** Torsten Schroeder, Guranda Chitadze, Justina Dargvainiene, Tjark Martens, Niklas Gebauer, Julia Frimmel, Monika Brüggemann, Thomas Valerius, Natalie Schub, Claudia D. Baldus, Christiane Pott, Frank Leypoldt, Klarissa Stürner, Friedrich Stölzel

**Affiliations:** 1 Department of Medicine II, Department of Haematology and Oncology, University Hospital Schleswig-Holstein Kiel, Kiel University, Kiel, Germany; 2 Institute of Clinical Chemistry, University Hospital Schleswig-Holstein Kiel, Kiel University, Kiel, Germany; 3 Department of Haematology and Oncology, University Hospital Schleswig-Holstein Lübeck, University of Lübeck, Lübeck, Germany; 4 Department of Neurology, University Hospital Schleswig-Holstein Kiel, Kiel University, Kiel, Germany

**Keywords:** CAR T-cell therapy, case report, CD19, ICANS, MNTS, neurotoxicity

## Abstract

We report a 63-year-old male patient with diffuse large B-cell lymphoma (DLBCL) who developed delayed-onset neurotoxicity on day +22 following CD19-directed CAR T-cell therapy with axicabtagene ciloleucel (axi-cel), after an initial episode of CRS and ICANS. The underlying disease had relapsed with secondary central nervous system (CNS) involvement, highlighting a high-risk setting for neurotoxicity. Unlike classical ICANS, the syndrome featured progressive gait ataxia and hypokinetic movement disturbances—clinical hallmarks of so-called movement and neurocognitive treatment-emergent adverse events (MNTs), a syndrome often referred to as parkinsonism due to characteristic features such as bradykinesia, rigidity, tremor, and cognitive slowing. To date, such MNTs have only been reported in patients receiving BCMA-targeted CAR T-cell products, primarily for multiple myeloma. Our report is, to the best of our knowledge, the first documented case of an MNT-like syndrome following CD19-directed CAR T-cell therapy. The patient’s symptoms evolved subacutely, in the absence of radiographic progression, infection, or lymphoma relapse. Immunophenotyping revealed activated effector-memory CD8^+^ T cells (HLA-DR^+^/CD38^+^/CD28^-^/PD1^+^) in peripheral blood, and predominantly CAR T cells in cerebrospinal fluid. Neurofilament light chain (NfL) levels rose significantly in serum and CSF, indicating neuroaxonal injury. Steroid therapy led to partial clinical improvement. Follow-up neuropsychological testing revealed persistent deficits in attention and processing speed. This case broadens the known neurotoxicity spectrum of CAR T-cell therapies and underscores the need for heightened clinical vigilance and refined diagnostic criteria beyond ICANS, even in CD19-targeted settings.

## Introduction

Chimeric antigen receptor (CAR) T-cell therapies have revolutionised the treatment of haematological malignancies and are increasingly applied in routine clinical practice. Alongside their remarkable efficacy, CAR T cells are associated with a broad spectrum of immune-related toxicities, of which immune effector cell-associated neurotoxicity syndrome (ICANS) is the most commonly recognised neurological complication (1, 2). ICANS typically presents within ten days post-infusion and is characterised by a stereotypical clinical course including writing disorder, encephalopathy, aphasia and seizures. Risk factors include disease burden, cytokine release syndrome (CRS), and the nature of the CAR construct (3). However, the operational definition of ICANS remains limited, and validated biomarkers to guide diagnosis or predict outcome are lacking.

In this context, atypical neurotoxic manifestations such as tumour inflammation-associated neurotoxicity (TIAN) (4) and movement and neurocognitive treatment-emergent adverse events (MNTs) (5–7) have emerged.

MNTs are rare, delayed-onset neurotoxic syndromes, with reported frequencies of up to 5%, and have so far been described exclusively in patients receiving BCMA-targeted CAR T-cell products (5–7). They typically arise after the resolution of CRS or ICANS, with a median onset of approximately 27 days post-infusion. Clinically, MNTs present with hypokinetic movement disorders such as bradykinesia, rigidity, tremor, and personality changes (5, 7). These manifestations extend beyond the established ICANS phenotype and challenge current diagnostic frameworks (6). Although the underlying pathophysiology remains poorly understood, sustained or compartmentalised immune activation—possibly involving clonally expanded CD8^+^ T cells—has been proposed, in contrast to the systemic cytokine-driven mechanisms typical of ICANS.

To our knowledge, no such cases have been reported following CD19-directed CAR T-cell therapy. We here present the first case of an MNT-like syndrome after axicabtagene ciloleucel (axi-cel) characterised by progressive ataxia. This report expands the known spectrum of CAR T-cell–associated neurotoxicity and underscores the need for heightened clinical awareness, refined diagnostic criteria, and deeper mechanistic insight into non-ICANS neurotoxic syndromes.

## Case description

A 63-year-old white male patient, married and academically educated, received axi-cel due to 2nd relapse of a diffuse large B cell lymphoma (DLBCL) Ann-Arbor stage IIa, which was initially diagnosed nine years earlier with cervical manifestations and relapsed four years later with secondary CNS involvement.

Following initial diagnosis, he received eight cycles of R-CHOP, followed by maintenance therapy with rituximab every 8 weeks for two years. The first relapse occurred four years later as part of a CNS manifestation of the lymphoma with histological evidence of DLBCL. The patient exhibited Wernicke’s aphasia with temporo-parietal localization on the left hemisphere. Four cycles of R-MATRIX followed by BCNU/thiotepa-based high-dose chemotherapy and autologous stem cell transplantation achieved a second complete remission (CR). Two and a half years after autologous transplantation a second relapse of the disease occurred, this time with localization on the right parietal region.

Reinduction therapy with two cycles of MATRIX chemotherapy was administered as bridging therapy, resulting in partial remission (PR), followed by standard lymphodepletion with fludarabine (30 mg/m²/day; total dose 189 mg) and cyclophosphamide (500 mg/m²/day; total dose 3180 mg) administered from day −5 to −3, and subsequent CAR T-cell therapy with axicabtagene ciloleucel at the planned full dose of 0.4–2 × 10^8^/kg body weight autologous CD19-directed CAR-positive T cells.

There was no barrier to therapy concerning cultural or financial aspects.

**Figure 1:**
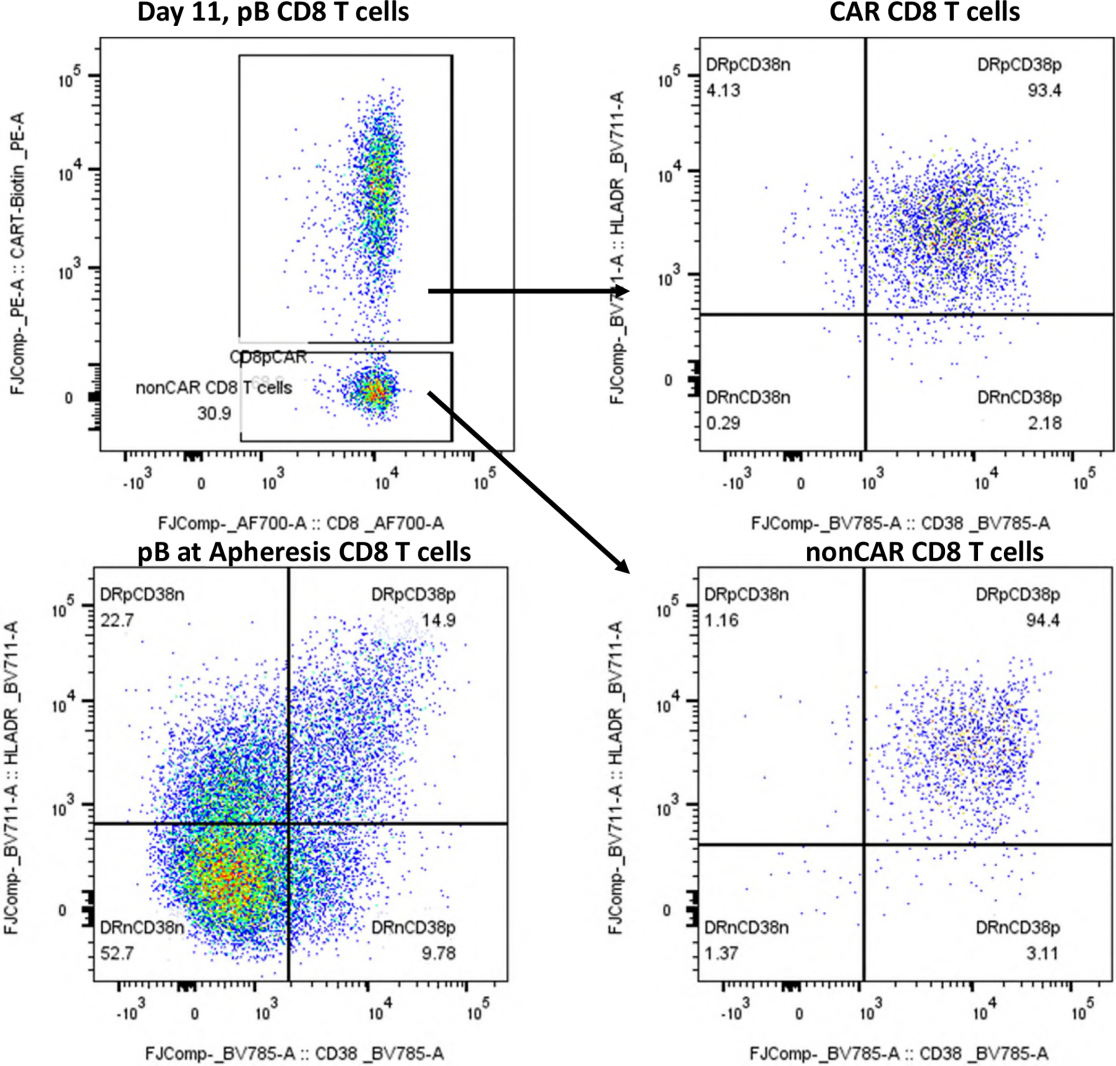
Immunophenotyping of peripheral blood CD8^+^ T cells at day +11 post-axi-cel infusion and at apheresis. Immunophenotypic analysis of peripheral blood CD8^+^ T cells by flow cytometry. Dot plots display expression of HLA-DR (y-axis) and CD38 (x-axis) on CAR-positive and non-CAR CD8^+^ T-cells at day +11 post-infusion, compared to CD8^+^ T cells at the time of apheresis. CAR T-cells were identified using an anti-CAR-specific detection reagent. At day +11, both CAR^+^ and non-CAR CD8^+^ T-cells exhibited a highly activated phenotype (HLA-DR^+^/CD38^+^), whereas CD8^+^ T cells from the apheresis product lacked activation markers. This pattern indicates an *in vivo* induction of activation following CAR T-cell infusion, likely influenced by the axi-cel product design (CD28 co-stimulatory domain).

**Figure 2:**
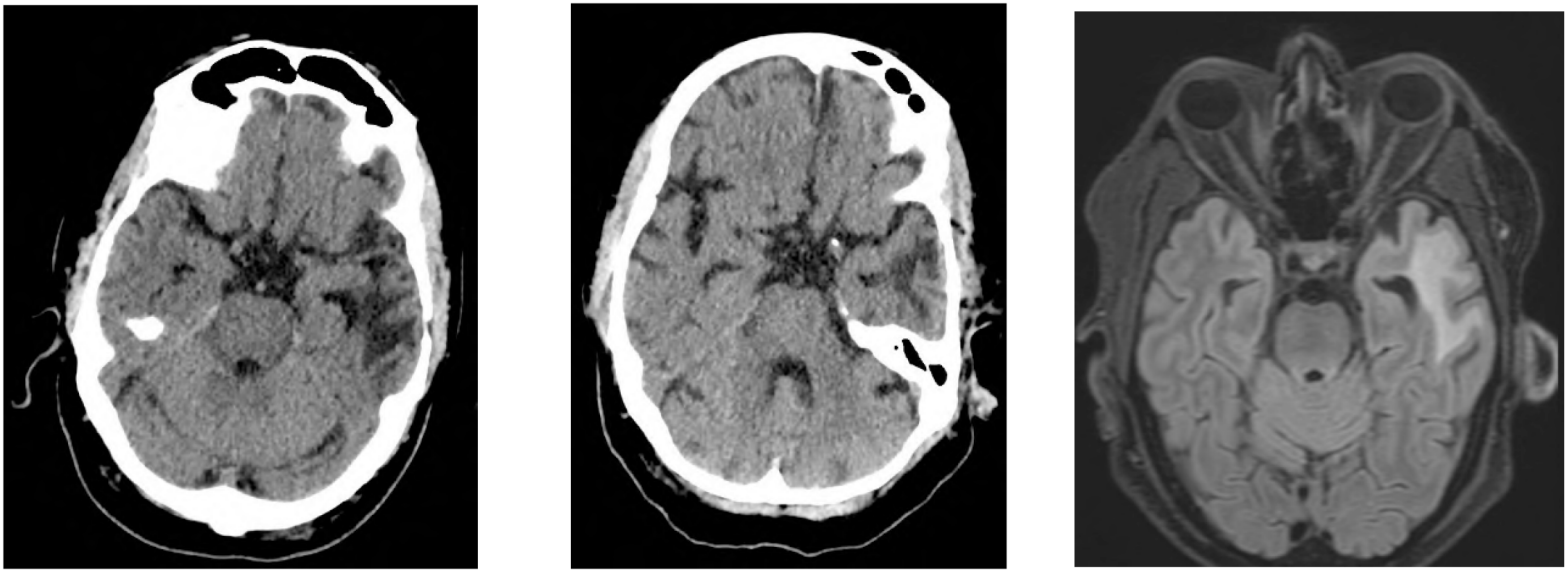
Cerebral imaging until day +28. **(A)** cCT day +1. **(B)** cCT day +5. **(C)** cMRI day +28. **(A)** On day +1, the patient developed ICANS; CT imaging shows a slightly regressive hypodensity in the right parietal area (right parietal = 2nd lymphoma recurrence). Known defect in the left temporoparietal area following tumour resection (=1st lymphoma recurrence), unchanged. **(B)** On day +5, due to progressive ICANS grade II-III with aphasia under dexamethasone, a repeat CT scan was performed. There was no indication of right-parietal progression as well as a constant parenchymal defect in the left temporal area due to previous tumour resection, nor were there any signs of intracerebral haemorrhage or early signs of infarction. **(C)** On day +16, the patient was discharged from the hospital, on day +22 he was readmitted because of MNTs and on day +28, follow-up staging imaging was conducted, which showed no new lymphoma manifestation on MRI.

Shortly after CAR T-cell infusion, the patient developed febrile enterocolitis during neutropenia as well as a grade 1 cytokine release syndrome (CRS). Additionally, he developed ICANS grade 2 with word-finding difficulties, reduced vigilance, and apraxia on day +1, requiring dexamethasone, which was then tapered. After symptom recurrence on day +4, dexamethasone was intensified and escalated to methylprednisolone due to further progression on day +6. This led to a resolution of symptoms allowing gradually tapering off the corticosteroids. Anticonvulsive prophylaxis with levetiracetam had been applied from day +1 to day +16. Peripheral blood immunophenotyping on day +11 showed 70% CAR+ T cells, 75% of which were CD8 +. Both CAR+ and non-CAR CD8+ T cells exhibited an activated effector memory phenotype (HLA-DR+/CD38+/CD28−/PD1+), absent at apheresis. In CD4+ T cells, 60% showed the same activation pattern (**Figure 1**). Neither on day +1, nor day +5 radiological signs of cerebral tumour progression or pseudoprogression were detected (**Figure 2**). On day +16, the patient was discharged from inpatient treatment without any neurological symptoms.

On day +22, he was readmitted to our department due to progressive gait instability and recurrent falls. Neurological examination was performed and revealed broad-based, ataxic gait. Furthermore, cerebellar signs were noted, most prominently limb and gait dysmetria, while nystagmus and dysarthria were absent. Muscle tone was globally increased. Deep tendon reflexes were normal and symmetrical. Sensory examination revealed no abnormalities. Oculomotor function was intact, with no gaze palsy or saccadic abnormalities. Overall, the neurological phenotype represented a mixed extrapyramidal–cerebellar syndrome with associated neurocognitive and motivational impairment, evolving subacutely after resolution of ICANS.

Over the following days, symptoms fluctuated and included reduced drive and severe appetite loss. Cerebral MRI showed no evidence of disease progression. A broad diagnostic work-up was performed, including repeated neurological examinations, exclusion of peripheral polyneuropathy, electroencephalography, lumbar puncture, and extensive infectious work-up. No relevant psychosocial stressors or familial predispositions were known.

Electroencephalography performed on day +29 revealed a pathological resting and activation EEG with a moderately slowed and modulated alpha background rhythm of approximately 8/s, accompanied by intermittent bifrontal and partially left frontotemporal slowing. No epileptiform discharges were detected, consistent with a bifrontal cerebral dysfunction.

Lumbar puncture on day +35 demonstrated clear cerebrospinal fluid with 3 leukocytes/µl and no erythrocytes, excluding relevant blood contamination. CSF chemistry showed normal glucose and lactate levels, mildly elevated total protein, and reduced immunoglobulin concentrations without evidence of intrathecal immunoglobulin synthesis. Cytopathological analysis revealed a nearly acellular cytoblock with only rare histiocytic cells and no evidence of lymphoma cells, including negative immunocytochemistry for CD20 and CD79a. Microbiological cultures of the CSF remained sterile, with no growth of bacteria or fungi. Flow cytometry and CAR T-cell–specific PCR analysis demonstrated the presence of CAR T cells in the CSF, accounting for the majority of detected leukocytes. In parallel, CAR T-cell PCR from peripheral blood on day +42 revealed low-level persistence with 5 vector copies/µl.

Retrospectively measured NfL levels analysed using single molecule array (Simoa) technology on the fully automated HD-X platform with a commercially available assay (NF-light Advantage V2, Quanterix, Billerica, USA) in serum and CSF remained stable between day +0 and day +11 (13.6–19.0 pg/ml, age adjusted Z-scores 0,44-1,37) but increased to 29.0 pg/ml (age adjusted Z-score 2,19) on day +28. In CSF, NfL was markedly elevated, measuring 2170.6 pg/ml on day +35 and 1957.4 pg/ml on day +96; serum NfL on day +96 was 34.3 pg/ml (age-adjusted Z-score 2,41) (8), consistent with central neuronal injury.

Treatment with methylprednisolone 1 g/day from day +35 to +39 led to significant clinical improvement. Steroid treatment was well tolerated and completed as planned. No other adverse events occurred during the corticosteroid course. The patient was discharged on day +45. Outpatient follow-up and rehabilitation led to gradual symptom improvement. On day +282, a comprehensive neuropsychological evaluation revealed subtle cognitive impairments. The test of attentional performance (TAP) (9) revealed impairments in attention (alertness without tone: 14th percentile, with tone: 4th percentile, phasic alertness: 1st percentile). Flexibility was reduced (27th percentile), with increased errors despite normal reaction times. The trail making test (TMT-A: 3rd percentile; TMT-B: 1st percentile) confirmed deficits in processing speed and cognitive flexibility.

In the Stroop test, reading (24th percentile) and naming (11th percentile) speeds were reduced, reflecting impaired cognitive control. While short-term and working memory were intact, learning, retention, and recognition were significantly impaired. Evaluation confirmed attentional slowing and omissions, alongside mnemonic deficits in learning and retaining a word list (**Supplementary Figure 1**).

**Figure 3:**
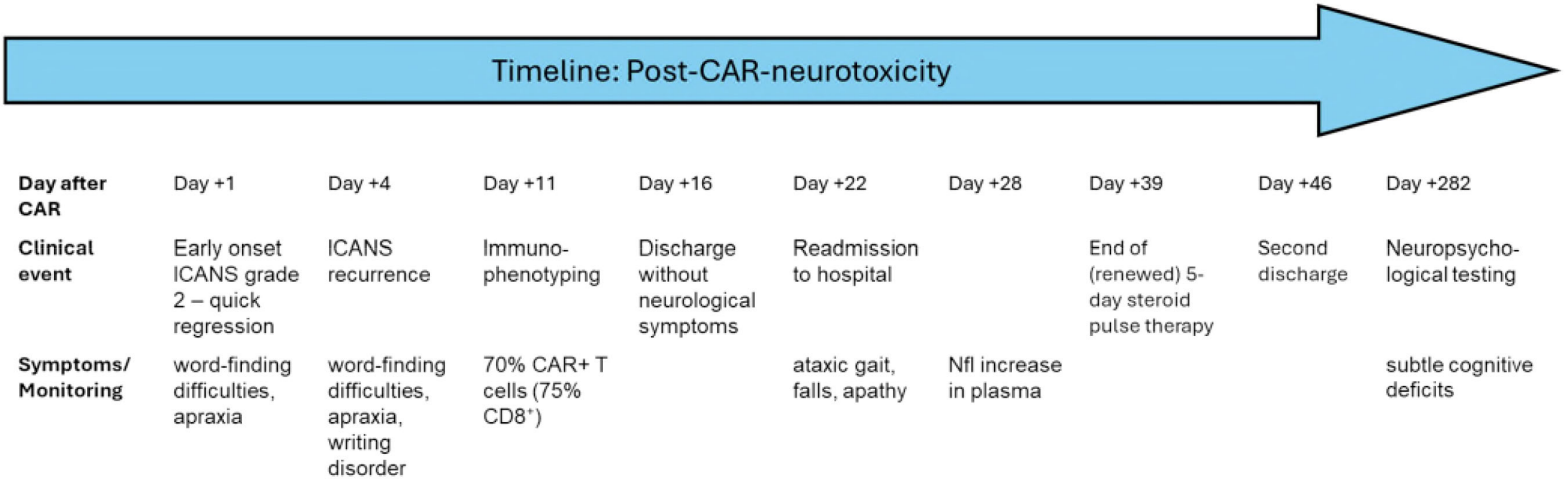
Timeline post-CAR-neurotoxicity. Clinical timeline of post-CAR T-cell neurotoxicity. The figure illustrates the temporal sequence of clinical events following CD19-directed CAR T-cell therapy with axicabtagene ciloleucel (axi-cel). After initial cytokine release syndrome (CRS) and immune effector cell-associated neurotoxicity syndrome (ICANS) occurring within the first week post-infusion, the patient experienced a second, delayed-onset neurological syndrome starting on day +22, characterised by ataxic gait and hypokinetic features. Cerebrospinal fluid (CSF) analysis on day +35 confirmed the presence of CAR T-cells and elevated neurofilament light chain (NfL) levels. High-dose corticosteroid therapy resulted in gradual clinical improvement. Follow-up neurocognitive testing on day +282, however revealed persistent but subtle deficits. The timeline highlights the diagnostic complexity and protracted course of non-ICANS neurotoxicity.

At 1.5 years post-therapy, the patient remained in remission with improved motivation and physical condition. The clinical picture strongly supports a secondary, distinct neurotoxicity pattern consistent with MNTs rather than recurrent ICANS (5, 6, 10). A timeline summarizing key events, diagnostics, interventions, and outcomes is shown in **Figure 3** and **Table 1**.

**Table 1:** Comparison between ICANS and non-ICANS-like toxicities.

Time point	Symptom type	Symptoms
Day +1	ICANS-like	Word-finding difficulties, decreased vigilance, apraxia
Day +4	ICANS-like	Recurrent word-finding difficulties, decreased vigilance, apraxia
Day +22	Non-ICANS-like	Mobility restriction, significant gait instability
Days +22 to +35	Non-ICANS-like	Gait ataxia, diminished drive, severe loss of appetite

## Discussion

Neurotoxicities following CAR T-cell therapy remain diagnostically and mechanistically complex. While ICANS is the most frequently encountered entity and typically presents with early monomorphic symptoms (1, 2, 11, 12), atypical neurotoxic syndromes require heightened clinical awareness and careful differential diagnosis. MNTs represent such a rare but distinct entity, previously described only in the context of BCMA-targeted CAR T-cell constructs (5).

In our case, the initial clinical presentation with word-finding difficulties, apraxia, and reduced alertness was consistent with ICANS and responded to corticosteroid therapy. However, from day +22 onwards, a second, delayed neurological syndrome emerged, characterized by ataxic gait, reduced motivation, appetite loss, and prominent extrapyramidal features including rigidity, bradykinesia, hypomimia, and postural instability. This evolution of symptoms, in the absence of radiological progression or infectious correlates, strongly suggested a secondary, delayed non-ICANS neurotoxicity within the spectrum of movement and neurocognitive treatment-emergent adverse events (MNTs) (6). While classical BCMA-associated MNTs have predominantly been described as parkinsonian syndromes, the mixed extrapyramidal–cerebellar phenotype observed in our patient indicates phenotypic heterogeneity rather than a distinct entity. We therefore classify this presentation as an atypical, MNT-like delayed non-ICANS neurotoxicity. A limitation of our clinical assessment is the lack of a baseline neurocognitive evaluation prior to CAR T-cell therapy.

Immunophenotyping on day +11 revealed a dominant CD8^+^ T-cell profile involving both CAR-positive and non-CAR T-cells, accompanied by an activated effector-memory phenotype. This profile is typical for early post-infusion phases of axi-cel, especially in patients with CRS and ICANS. Notably, this CD8^+^-dominant immune composition persisted beyond the ICANS phase. By day +42, CAR T vector copy numbers in peripheral blood had decreased substantially (5 copies/µl), yet no corresponding rebalancing in the CD4/CD8 ratio was observed. This skewing suggests ongoing immune activation, either from persisting CAR T-cells or bystander CD8^+^ expansion. In the cerebrospinal fluid on day +35, only three leukocytes per µl were detected, 80% of which were CAR T-cells. This contrasts with prior reports where clonally expanded non-CAR CD8^+^ T cells dominated the CSF in BCMA-associated MNTs (10). These findings underscore that the pathophysiology of these atypical neurological manifestations remains incompletely understood. While the clinical phenotype in this case clearly resembles an MNT-like syndrome, it is conceivable that multiple, distinct immunopathological processes may give rise to similar presentations.

Neurofilament light chain levels, although not validated for this indication, showed a delayed rise in plasma on day +28 and were markedly elevated in the CSF on days +35 and +96. While such findings support the presence of neuronal injury, the clinical utility of NfL in CAR T-cell–associated neurotoxicity remains uncertain due to the lack of validation in this rare context.

As the patient had symptomatic secondary CNS lymphoma, comparison with MNT cases in multiple myeloma without CNS involvement is limited. Malignant CNS manifestations may pose general risk of neurological adverse events, though this remains unproven. The patient’s symptoms during the second neurological phase also necessitated consideration of tumour inflammation-associated neurotoxicity (TIAN) as a differential diagnosis, particularly in light of his history of CNS lymphoma and initial febrile presentation. TIAN is a recently described form of neurotoxicity associated with intraparenchymal or meningeal tumour infiltration and is typically divided into type 1 (mass effect and edema-driven) and type 2 (cytokine-mediated, inflammation-driven without focal structural changes) (4). However, the absence of radiological evidence of parenchymal edema or mass effect, the lack of localised symptoms such as neglect or hemianopsia, and the fluctuating clinical course with delayed onset argue against TIAN as the primary mechanism. Some overlap in symptomatology and steroid-responsiveness may exist (**Supplementary Figure 2**).

Altogether, no other predisposing factors or diagnoses were identified to explain the clinical presentation.

In summary, this case illustrates that MNTs may not be exclusive to BCMA-directed CAR T-cell products and can occur after CD19-targeted therapy. The clinical and immunological features observed here expand the current understanding of CAR T-cell–associated neurotoxicity and underscore the need for ongoing mechanistic studies to distinguish ICANS from other entities such as MNTs and to develop targeted monitoring and intervention strategies (7).

## Patient perspective

After initial hospital discharge the patient described that the first inpatient stay for CAR-T cell therapy had been very exhausting. He could not clearly recall the details of the time spent at home before his readmission. Assuming that his nutritional intake at home had been adequate and that he had moved sufficiently firstly after second readmission. Asked at follow-up appointments more than six months when looking back, he still could not remember the specific details from that period but noted a slow and steady improvement in both cognition and mobility. In addition, he gradually began to regain some motivation and desire on the one hand for physical activity, and on the other hand for engagement in everyday social interactions.

## Data Availability

The original contributions presented in the study are included in the article/**Supplementary Material**. Further inquiries can be directed to the corresponding author.
