# Supplementary material for: Non-ICANS neurotoxicities CD19-directed CAR T-cell therapy and the emergence of movement and neurocognitive treatment-emergent adverse events: a case report
Source: Front Immunol. 2026 Feb 16;17:1749587. doi: 10.3389/fimmu.2026.1749587 (PMC12950781; doi:10.3389/fimmu.2026.1749587)
Supplement: Supplementary file 1 [file DataSheet1.pdf]

Supplementary Figures

Supplementary Figure 1: Neurocognitive testing on day +282

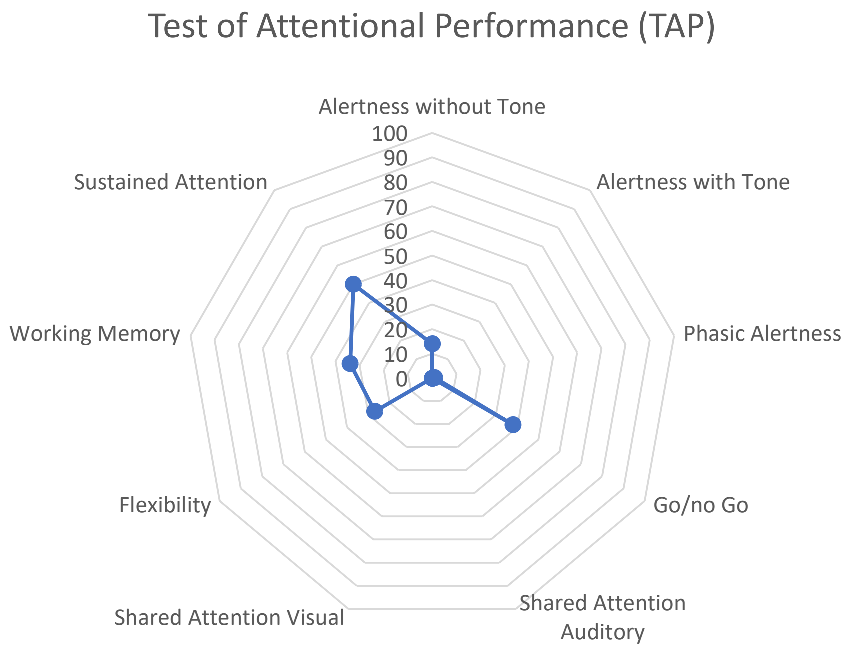

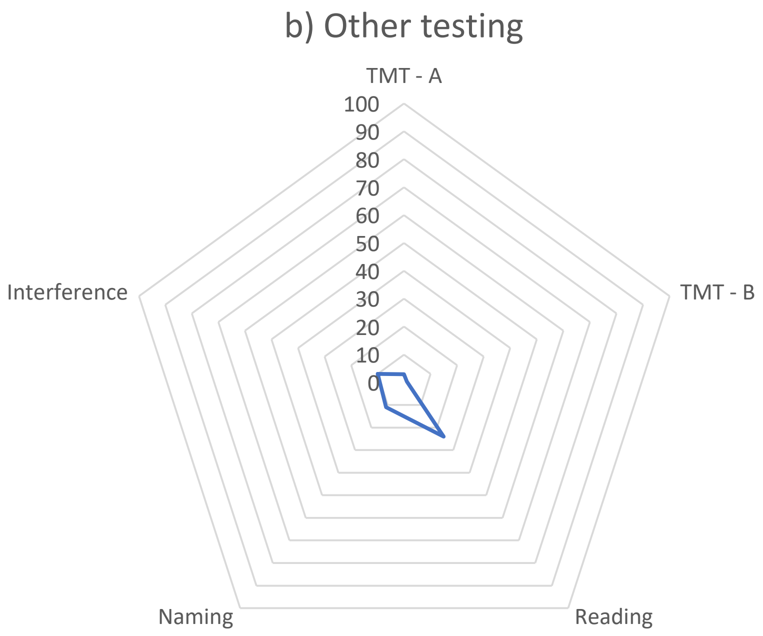

Supplementary Figure 1 legend:

Results of the testing of neurocognitive functions in percent compared to the normative sample. The test was conducted on day +282 after CAR T-cell therapy.

Fig. a) underlines an impairment in alertness and shared attention while results in reaction inhibition (Go/no Go) and sustained attention show ordinary results.

Fig. b) illustrates the results of the trail making test (TMT-A/-B) displaying an impairment in neuropsychologic functions. Reading seems to be hardly worsened. However, naming seemed to be difficult yet, underlying lymphoma localization that has been in speech areas. Additionally, there was a deterioration in the interference test, in which words are presented in different colors and the color of the word must be named, rather than reading the word itself. This test assesses executive functions, attention, and processing speed.

Supplementary Figures 2A, 2B, and 2C: Different forms of neurotoxicity post CAR T-Cell therapy

2A

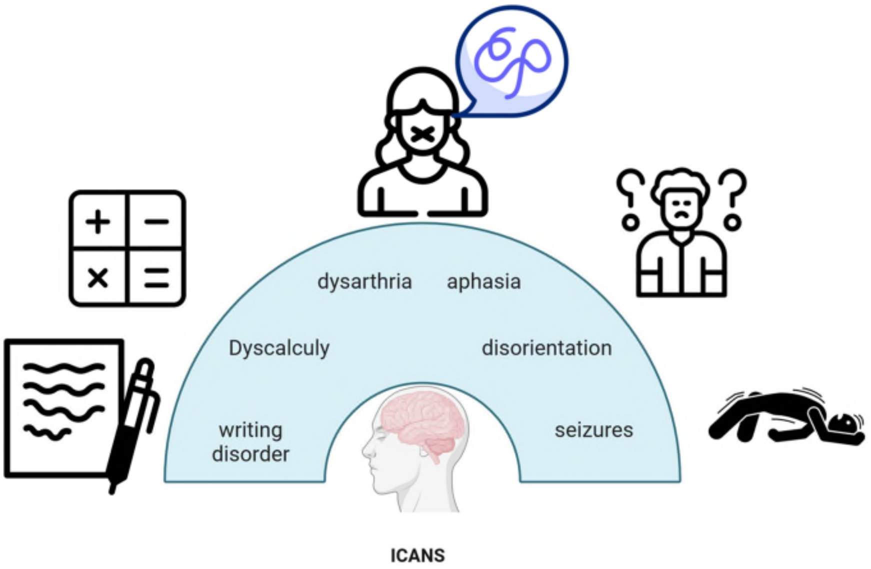

2B

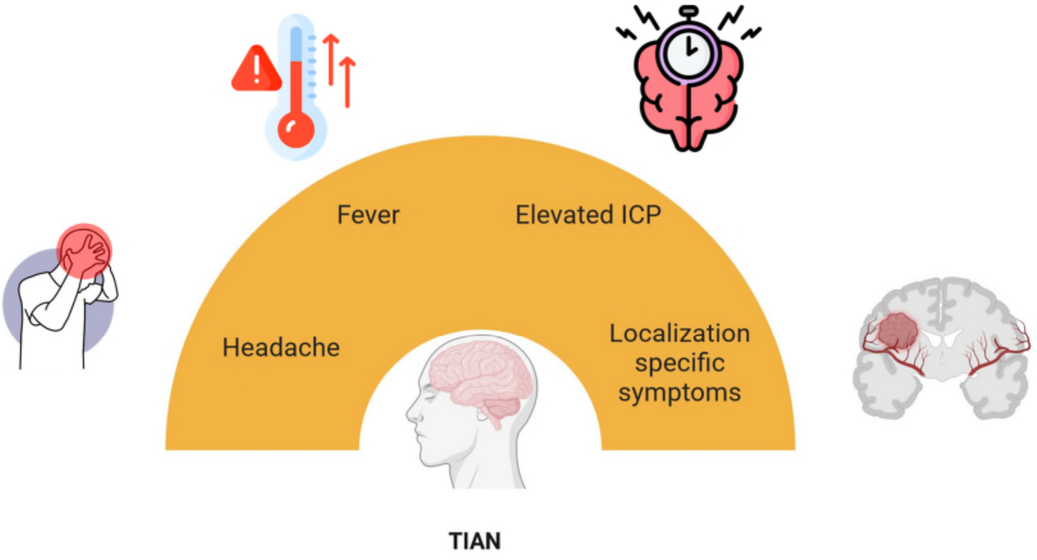

2C

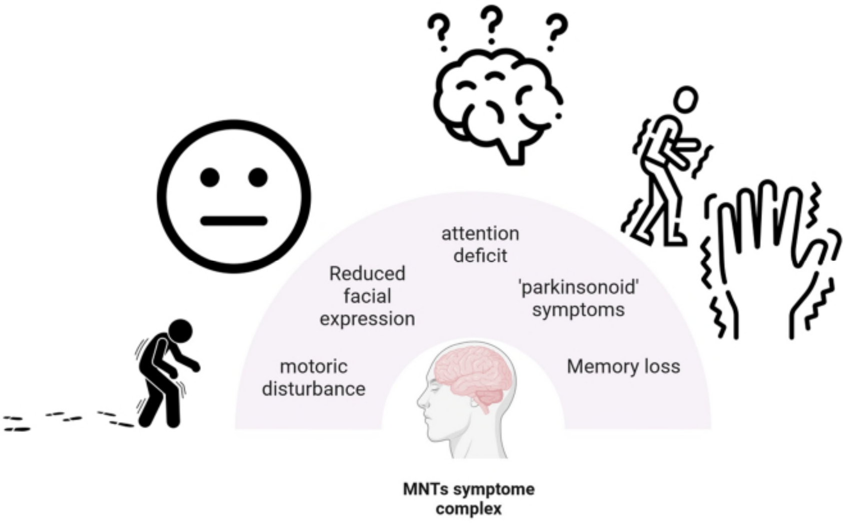

Supplementary Figure 2 Legend

Supplementary Figure 2A depicts various forms of neurotoxicity associated with CAR T-cell therapy. In the first segment, ICANS (Immune Effector Cell-Associated Neurotoxicity Syndrome) is depicted with a range of cognitive and neurological symptoms. These include disorientation, represented by a perplexed individual, aphasia, dysarthria, writing disorders, and dyscalculia. Seizures may occur typically in higher-grade ICANS.

Supplementary Figure 2B, TIAN (Tumour Inflammation-Associated Neurotoxicity) is characterized as another acute CAR T-cell-associated neurotoxicity. Characteristic symptoms are headache, fever, and symptoms that are associated with the tumour localization. An elevation of intracranial pressure (ICP) is also possible. In contrast to ICANS and MNTs, TIAN is a neurotoxicity that is exclusively described in intracranial tumour localizations, whereas ICANS and MNTs may occur regardless of tumour localization.

Supplementary Figure 2C, MNTs (Movement and Neurocognitive Treatment-Emergent Adverse Events), highlights motor and cognitive symptoms. Exemplary Parkinsonoid-like symptoms, such as gait disturbances, imbalance, tremor, flat affect, and reduced facial expression, may occur. An attention deficit is also described.
