# Supplementary material for: Non-ICANS neurotoxicities CD19-directed CAR T-cell therapy and the emergence of movement and neurocognitive treatment-emergent adverse events: a case report
Source: Front Immunol. 2026 Feb 16;17:1749587. doi: 10.3389/fimmu.2026.1749587 (PMC12950781; doi:10.3389/fimmu.2026.1749587)
Supplement: Supplementary file 2 [file DataSheet2.pdf]

CARE Checklist of information to include when writing a case report

CARE case report guidelines

Topic	Item	Checklist item description	Reported on Line
Title	1	The diagnosis or intervention of primary focus followed by the words "case report"	Title
Key Words	2	2 to 5 key words that identify diagnoses or interventions in this case report, including "case report"	56
Abstract (no references)	3a	Introduction: What is unique about this case and what does it add to the scientific literature?	44-45
	3b	Main symptoms and/or important clinical findings	39-40
	3c	The main diagnoses, therapeutic interventions, and outcomes	40-41
	3d	Conclusion—What is the main "take-away" lesson(s) from this case?	54-55
Introduction	4	One or two paragraphs summarizing why this case is unique (**may include references**)	94-95
Patient Information	5a	De-identified patient specific information.	99
	5b	Primary concerns and symptoms of the patient.	112–115, 123–126
	5c	Medical, family, and psycho-social history including relevant genetic information	99
	5d	Relevant past interventions with outcomes	103-110
Clinical Findings	6	Describe significant physical examination (PE) and important clinical findings.	115–117
Timeline	7	Historical and current information from this episode of care organized as a timeline	289, fig. 3
Diagnostic Assessment	8a	Diagnostic testing (such as PE, laboratory testing, imaging, surveys).	118–128, 150–155
	8b	Diagnostic challenges (such as access to testing, financial, or cultural)	112
	8c	Diagnosis (including other diagnoses considered)	129-133
	8d	Prognosis (such as staging in oncology) where applicable	100
Therapeutic Intervention	9a	Types of therapeutic intervention (such as pharmacologic, surgical, preventive, self-care)	103-112, 116-119, 140-141
	9b	Administration of therapeutic intervention (such as dosage, strength, duration)	103-112, 116-119, 140-141
	9c	Changes in therapeutic intervention (with rationale)	140-142
Follow-up and Outcomes	10a	Clinician and patient-assessed outcomes (if available)	143 -155
	10b	Important follow-up diagnostic and other test results	143-155
	10c	Intervention adherence and tolerability (How was this assessed?)	141
	10d	Adverse and unanticipated events	125-127
Discussion	11a	A scientific discussion of the strengths AND limitations associated with this case report	158 and following
	11b	Discussion of the relevant medical literature **with references**.	159-196
	11c	The scientific rationale for any conclusions (including assessment of possible causes)	171-196
	11d	The primary "take-away" lessons of this case report (without references) in a one paragraph conclusion	202-203
Patient Perspective	12	The patient should share their perspective in one to two paragraphs on the treatment(s) they received	207-215
Informed Consent	13	Did the patient give informed consent? Please provide if requested	Yes ☒ No ☐
